# A Mobile Health Breast Cancer Educational and Screening Intervention Tailored for Low-Income, Uninsured Latina Immigrants

**DOI:** 10.1089/whr.2020.0112

**Published:** 2021-08-16

**Authors:** Maria De Jesus, Shalini Ramachandra, Alexis De Silva, Shirley Liu, Ethan Dubnansky, Kingsley Iyawe, Astrid Jimenez, Laura Logie, M.C. Jackson

**Affiliations:** ^1^ Center on Health, Risk, and Society, School of International Service, American University, Washington, District of Columbia, USA.; ^2^ Department of Mathematics and Statistics, American University, Washington, District of Columbia, USA.; ^3^ Department of Mathematics and Statistics, Connecticut College, New London, Connecticut, USA.; ^4^ Nueva Vida, Alexandria, Virginia, USA.

**Keywords:** educational intervention, mHealth, breast cancer, screening mammography, Latina immigrants

## Abstract

***Objective:*** To investigate the efficacy of mobile health (mHealth) intervention strategies that delivered either personalized, culturally, and linguistically tailored cell phone voice messages or text messages related to breast cancer and prevention, compared to the control group, to determine which strategy is more likely to increase breast cancer knowledge and screening mammography among low-income Latina immigrants.

***Methods:*** This randomized controlled trial assigned 256 Latina immigrants 40 years of age or older to one of three groups: an automated cell phone voice message group, an automated text message group, or the control group (mail). The mHealth intervention employed a comprehensive approach that included breast cancer and prevention education and free mammography screening. Outcome measures included knowledge of breast cancer and breast cancer prevention, and adherence to screening mammography.

***Results:*** There was a general increase in breast cancer knowledge after the educational intervention for all the groups [*p* = 0.01, *t*(199) = 3.996]. Knowledge increase and mammography adherence did not differ based on group.

***Conclusion:*** More important than the actual method of communication is *how* breast cancer and prevention messages are constructed, *who* the messenger is, and the enabling factors that facilitate screening adherence. A breast cancer preventive intervention program that is personalized, culturally and linguistically tailored, and offers a free or low-cost mammogram holds promise to be an effective method in reaching an underserved Latina population with a high breast cancer burden.

## Introduction

Ethnic/racial disparities in breast cancer and breast cancer screening persist. While the breast cancer incidence rate remained stable in non-Hispanic white women from 2005 to 2015, it increased among Hispanic women or Latinas (0.4% annually),^1^ who comprise the largest ethnic minority and one of the fastest growing ethnic groups in the United States.^2^ Despite a lower overall incidence rate of disease (99.1 for Hispanic women vs. 131.3 per 100,000 non-Hispanic white women), breast cancer is the leading cause of cancer death among Latinas.^3^ Hispanic women are also disproportionately diagnosed with breast cancer at later stages compared to non-Hispanic white women.^1,4^

Data also demonstrate that Latinas, especially those who are uninsured, have relatively lower rates of screening mammography participation and have delayed follow-up of abnormal screening results or self-discovered breast abnormalities.^5^ The 2015 prevalence of mammography in the past 2 years was lower among Hispanic (61%) than among white (65%) and black (69%) women 40 years of age and older.^5^ In addition, in 2015, uninsured women (31%) and recent immigrants (46%) reported the lowest prevalence of mammography use in the past 2 years.^5^

The US Preventive Services Task Force (USPSTF) currently recommends biennial screening mammography for women between 50 and 74 years of age. Women may choose to begin screening between the ages of 40 and 49 years if they perceive potential benefits exceed potential harms.^6^ The American Cancer Society recommends regular yearly screening mammography for women beginning at the age of 45 and then change to biennial screening at age 55.^7^

Numerous barriers to breast cancer screening among Latina immigrants have been identified through previous research, including access barriers such as lack of health insurance, cost of a mammogram, inability to miss work or ask permission to miss work, lack of immigration documents or legal status, lack of a usual source of care, and lack of needed resources such as an interpreter and transportation.^8–12^

Mobile health (mHealth), defined as the delivery of health care information or services through mobile communication devices, carries important implications for reducing barriers to health care, health knowledge, behavior, and outcomes, including breast cancer prevention, while reducing the costs of health care.^13–16^ mHealth interventions have been found to be effective in improving patient compliance with medical recommendations; increasing satisfaction among patients; improving attendance rates at scheduled appointments; promoting healthy behavioral changes; reducing the costs for staff follow-up; and lessening the time from diagnosis to treatment.^13–16^

In a previous study with Korean immigrant women, a mobile phone app-based intervention combined with health navigator service showed a greater change on scores of knowledge of breast cancer and screening guidelines, as well as a higher proportion of completed mammograms by the 6-month follow-up compared with the control group.^17^ A recent systematic review that assessed the effect of text messaging interventions on screening for various cancers demonstrated a moderate increase in screening rates for breast and cervical cancer and a small effect on colorectal cancer screening.^18^

Despite the evidence highlighting the effectiveness of mHealth interventions in promoting behavioral change in various clinical settings, there is a paucity of data on the impact of these interventions on breast cancer knowledge and screening behavior, particularly among low-income immigrant women. This study, therefore, filled a critical gap in the literature by examining the effectiveness of an mHealth intervention among a low-income, mostly uninsured, and underscreened Latina immigrant population.

Given the high use of mobile phone technology among Hispanic women,^19^ the main study objective of the three-arm randomized control trial (RCT) was to investigate the efficacy of mHealth intervention strategies to determine which strategy was more likely to increase breast cancer knowledge and screening mammography among low-income Latina immigrants. We also assessed which factors (*i.e.*, predisposing, enabling, or reinforcing) would be most predictive of the dependent factors. The four hypotheses were as follows:

(1) The intervention group who received automated cell phone voice messages would have the highest proportion of mammography adherence, then the text message group, and finally the mail group;
(2) There would be a general increase in breast cancer and breast cancer prevention knowledge, measured by an increase in score from presurvey to postsurvey;
(3) The intervention group who received automated cell phone voice messages would have the highest level of knowledge of breast cancer and breast cancer prevention postintervention, followed by the text message intervention group, and finally the mail group; and
(4) The predisposing factors (*e.g.*, age and years in the US education level) and environmental factors (*e.g.*, health insurance status) would be predictive of screening mammography, given the results of previous research.^8–10^

## Methods

### Recruitment strategy and sample

From April 2015 to May 2017, we recruited 300 participants from community health centers, beauty salons, and churches in the Greater Washington D.C. Metropolitan area. Furthermore, members of the study team also distributed flyers in the Spanish language at the various sites and announced the study in the Latino ethnic press and radio stations. Trained bilingual and bicultural patient navigators described the study and obtained informed consent and contact information from each participant.

Eligibility criteria to participate in the study included the following: (1) Hispanic ethnicity; (2) 40 years of age or older; (3) overdue for a routine mammogram (>2 years) or never had one per self-report and confirmed through a medical chart review; (4) no personal history of breast cancer; (5) no presenting cancer symptoms; (6) own a cell phone number where she could be reached; and (7) knew how to receive and accept text and voice messages on her cell phone. Given that at the time this study was conducted, the American Cancer Society (ACS) recommended that women begin regular, annual screening mammography at age 40 years, the eligible age to participate in the study began at age 40.^20^ The study protocol was approved by the American University Institutional Review Board.

**FIG. 2.**
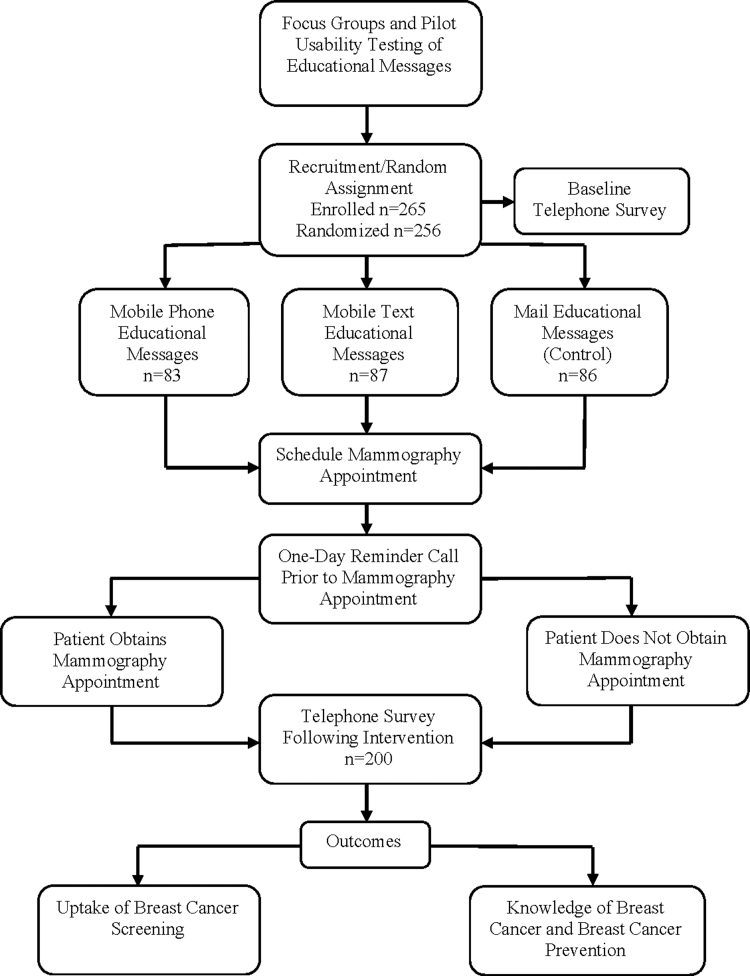
Study flow chart.

The total number of participants who ultimately enrolled in this study was 265. Nine individuals were not assigned to an educational intervention strategy due to missing data. Using a computer-generated algorithm, we randomly assigned 256 individuals to the control and two intervention groups (Fig. 2). A total of 200 individuals completed the postsurvey. Incorporating the ACS recommendation into this study, mammography adherence was defined as completing a mammogram postintervention to be current with the annual breast cancer screening per guidelines at the time of the study.

### Educational intervention

Drawing on a community-based participatory research approach, we designed and implemented this study with staff members from our community partner, *Nueva Vida*, a nonprofit, community-based organization providing free cancer screenings (through affiliated screening sites) and comprehensive and culturally competent services to Latinas with cancer or at risk for developing cancer. We also formed a community advisory board to provide guidance throughout the process of study development, execution, and dissemination of research findings.

The three-arm RCT compared the efficacy of (1) cell phone voice messages; (2) text messages; and (3) mail (control group) related to breast cancer and prevention to identify the best strategy to increase breast cancer knowledge and screening mammography. We conducted formative research, which guided the message development and included two rounds of focus group pilot testing with participants who had similar demographic characteristics to the target sample. Across all three methods of communication, the same breast cancer and screening information was tailored to a low literacy level. All the messages were also personalized, that is, they addressed the participant by her first name, and included information based on the gaps in women's knowledge of breast cancer and breast cancer prevention, identified during the formative phase.

**FIG. 1.**
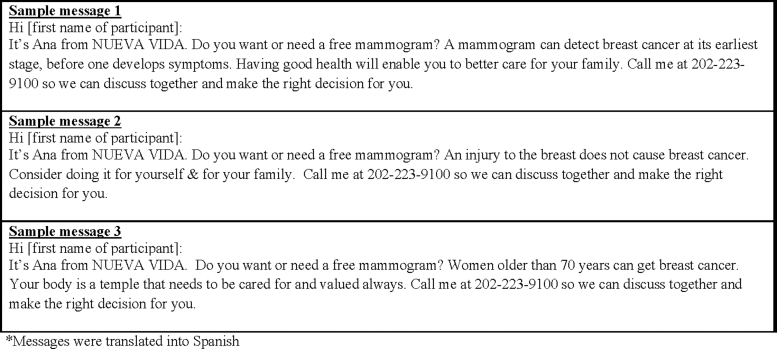
Examples of personalized, linguistically* and culturally tailored educational messages.

In addition, the messages were culturally tailored (*i.e.*, infused with salient themes from the focus groups such as family and faith) and linguistically appropriate (*i.e.*, translated into Spanish). We also incorporated a model of shared decision making into the messaging (Fig. 1). The messenger was a patient navigator from *Nueva Vida* who was considered by the women to be a trusted and credible source of information. Focus group data revealed that the appropriate number of messages per week was two over a period of 1 month, with a length of ∼15 seconds each.

Once participants were randomly assigned into groups, the educational intervention began (Fig. 2). The first step was a brief sociodemographic survey followed by a presurvey assessing knowledge of breast cancer and breast cancer prevention, administered over the phone by a *Nueva Vida* patient navigator.

The first intervention group received two personalized, culturally and linguistically tailored automated voice educational messages per week for 1 month, which were recorded by a *Nueva Vida* patient navigator. The second intervention group received two personalized, culturally and linguistically tailored automated text messages per week for a month, which were sent by a patient navigator. The mail group (control group) received the same messages through a letter sent in the mail by a patient navigator.

The patient navigators employed a computer telephony system called *Healthwave Phonetree*, a competitive web-based automated messaging service, to send the automated voice and text messages to the study participants; and track other information (*e.g.*, when the message was delivered, whether the participant received the message; and whether the participant viewed or listened to the message). Message tracking information indicated that all the participants viewed or listened to the text or voice message, respectively.

Following the receipt of a total of eight educational messages over a 1-month period for the two intervention groups, participants called to schedule their own mammography screening appointment based on their preferred dates and times. The screenings were provided for free by affiliated screening mammography sites. To further ensure accessibility, *Nueva Vida* also provided transportation to the screening sites on Saturdays for women who needed this resource. 

All study participants received an automated reminder phone call 1 day before their screening appointment. Within a 2-week window following the date of the screening appointment, a patient navigator administered a postsurvey to participants through telephone, regardless of whether they went to their screening appointment or not. The postsurvey was identical to the presurvey. Each participant received a monetary incentive for completing each survey.

### Sample size

A power analysis was calculated using G-power.^21^ The results indicated that a target study recruitment of 300 participants, with ∼100 in each arm, would provide over 95% power with an effect size of <3% difference between study groups in proportion of women who are screened for the three groups, with alpha 0.05 using an *F* test or a chi-square test.

### Outcome measures

Screening mammography outcome (yes/no) was the primary outcome measure, extracted from medical charts. The secondary outcome measure, knowledge of breast cancer and breast cancer prevention, was assessed before and after the educational intervention. The presurvey and postsurvey comprised 13 true-or-false items with a “Not Sure” option, addressing information gaps and misconceptions about breast cancer and breast cancer prevention, as identified in the focus groups with a subsample of women during the formative phase of the intervention. Examples of items were as follows: *A strong blow to a breast can cause breast cancer over time; Most of the lumps found in the breasts are cancerous;* and *A mammography is a test that detects breast cancer in its earliest stage, before symptoms develop*. 

The knowledge prescore and postscore was computed, respectively, by the number of items the participant answered correctly. Incorrect and “Not Sure” answers were given zero points, while a correct answer was given one point; the highest total score that an individual could receive was 13. Participants' overall scores on the survey, with possible scores ranging from zero to 13, were then used for statistical analyses. The internal consistency for this sample was good (alpha = 0.85 for the pretest and alpha = 0.87 for the posttest).

### Statistical methods

The significance level *α* = 0.05 was used for all the tests to compare with the obtained *p*-value, except for the regressions, which had a significance level of *α* = 0.10. All assumptions were reasonably met for every test, and all analyses were completed with the use of Stata 16.^22^

To address the multiple components of the study objective, various analyses were performed. A cross-tabulation and chi-square goodness-of-fit test were utilized to compare mammography adherence per educational intervention group (*i.e.*, voice message, text message, and mail). A matched pairs *t*-test was completed. The null hypothesis for the matched pairs *t*-test was that there was no difference between presurvey and postsurvey score as opposed to the alternative hypothesis of an increase in score (post minus pre). An analysis of variance (ANOVA) test to examine if there was a difference in survey score change based on intervention group was run. Correlation tests and various models of multiple regression were used to test and compare the statistical significance and impact of the factors accounted for within the study. 

Binary logistic models and multiple regression models were run to examine the variance within the participants' mammography adherence and level of breast cancer knowledge before the intervention, both stratified by intervention group and nonstratified. In the binary logistic models, since mammography adherence is measured as a categorical variable, the Cox and Snell *R*-square estimate were used to examine whether the full model is better in explaining the variance in the response variable than the null model. The odds ratio (OR) was reported for each explanatory variable within each model. In addition, the standardized coefficients were reported for the multiple regression models.

## Results

### Sociodemographics of the sample

Tables 1 and 2 summarize the sociodemographic information for continuous and categorical variables, respectively. The mean age of all participants was 48.44 years (standard deviation [SD] 7.84). On average, the participants had lived in the United States for 14.32 years (SD 8.29). The majority of the participants were either from Central America (60.0%) or South America (20.8%). In terms of educational background, 56.2% of the participants had received no education or an elementary school education. Most of the participants had low English proficiency with 67.9% reporting that they are not able to hold a conversation in English at all (38.1%) or not much (29.8%). With regard to employment, 66.9% were unemployed (33.3%) or employed on a part-time basis (33.6%).

**Table 1.** Numerical Demographic Characteristics of Latina Participants for Quantitative Variables

	Minimum	Maximum	Median	Mean	Standard deviation
Age	39	86	46	48.44	7.837
How many years have you lived in the United States?	0	40	14	14.32	8.288
How far away is the mammography site from your home? (miles)	0.8	69.8	13.3	16.832	11.9251
Presurvey score	1	13	8	7.86	2.378
Postsurvey score	3	13	9	8.61	2.538

**Table 2.** Frequency Demographic Characteristics of Latina Participants for Categorical Variables

Characteristic	Sample size	Percentage
How often do you ask for help when it comes to getting medical materials explained?		
Always	91	34.3
Often	31	11.7
Sometimes	51	19.2
Rarely	32	12.1
Never	43	16.2
N/A	17	6.4
How confident are you about filling out medical forms in English?		
Not confident	140	52.8
Somewhat confident	72	27.2
Confident	35	13.2
N/A	18	6.8
How often do you struggle to find out more about your medical condition?		
Always	85	32.1
Often	29	10.9
Sometimes	67	25.3
Rarely	39	14.7
Never	28	10.6
N/A	17	6.4
Could you hold a conversation in English?		
Not at all	101	38.1
Not much	79	29.8
Somewhat	45	17.0
Very well	21	7.9
N/A	19	7.2
Could you read a newspaper or book in English?		
Not at all	109	41.1
Not much	72	27.2
Somewhat	42	15.8
Very well	25	9.4
N/A	17	6.4
During your visit to the doctor, what language do you most often speak?		
English	9	3.4
Spanish	198	74.7
Both	41	15.5
N/A	17	6.4
How comfortable are you discussing health issues with your doctor?		
Not at all	6	2.3
Only a little	24	9.1
Somewhat comfortable	98	37.0
Very comfortable	114	43.0
N/A	23	8.7
Where were you born?		
Central America	159	60.0
South America	55	20.8
Mexico	23	8.7
Dominican Republic	5	1.9
N/A	23	8.7
Do you have health insurance?		
Yes	39	14.7
No	209	78.9
N/A	17	6.4
Employment status		
I am in school	2	0.8
Unemployed for >12 months	77	29.1
Unemployed for 12 months or less	11	4.2
Part-time employed	89	33.6
Full-time employed	71	26.8
N/A	15	5.7
Highest education degree		
No education	93	35.1
Elementary	56	21.1
High school	79	29.8
College/grad	22	8.3
N/A	15	5.7
Where did you receive information about breast cancer prevention?		
Doctor	69	26.0
Family/friends	35	13.2
Media	54	20.4
Church	24	9.1
Other	65	24.5
N/A	18	6.8
In general, how would you describe your health?		
Excellent	12	4.5
Good	99	37.4
Somewhat good	110	41.5
Poor	26	9.8
N/A	18	6.8
In general, how would you rate the medical or health quality you have received?		
Excellent	47	17.7
Good	105	39.6
Regular	55	20.8
Poor	9	3.4
No health care received	26	9.8
N/A	23	8.7
How much do you think you know about “Affordable Care?”		
A lot	8	3.0
More or less	53	20.0
Little	95	35.8
Nothing	92	34.7
N/A	17	6.4
Is there a place where you often go when you feel sick or need help?		
Yes	4	1.5
No	244	92.1
N/A	17	6.4
Do you have access to transportation?		
Yes	195	73.6
No	55	20.8
N/A	15	5.7

N/A, no answer.

In terms of current health status and health insurance status, respectively, 51.3% of the participants reported to be in somewhat good (41.5%) or poor health (9.8%), and the majority (78.9%) were uninsured. Most of the participants (92.1%) also reported not having a usual place of care. The groups were not different in these baseline characteristics, as indicated by the statistical nonsignificant *t* test and chi-square test results. Table 1 summarizes a five-number summary (minimum, maximum, median, mean, and SD) for all quantitative variables used in the analysis. The variables presurvey score and postsurvey score are measured out of 13, with points being given by correct answers chosen on the survey.

Table 2 summarizes the sample sizes of the categories per variable as well as the related percentages. For all questions, no answer (N/A) refers to the answer options not being applicable to the participant or that the participant chose not to answer the question.

### Hypothesis 1: screening mammography outcome by educational intervention group

Table 3 summarizes the results of a cross-tabulation between each educational intervention group and whether or not individuals received a screening mammography. The group receiving the intervention through mail had the greatest adherence to screenings (67.4%), and the overall mammography adherence across all three groups was 62.9%.

**Table 3.** Cross-tabulation of Mammography Outcome and Educational Intervention Group

	Mail	Phone	Text	Total
Received a mammogram?				
No	28 (32.6%)	34 (41.0%)	33 (37.9%)	95 (37.1%)
Yes	58 (67.4%)	49 (59.0%)	54 (62.1%)	161 (62.9%)
Total	86 (100%)	83 (100%)	87 (100%)	256 (100%)

The results of the chi-square goodness-of-fit test for mammography adherence and intervention group reveals that the *p*-value was 0.518 with *χ*(2) = 1.317. Contrary to the first hypothesis, there is insufficient evidence to suggest that mammography adherence differed based on the intervention group.

### Hypothesis 2: increase in knowledge of breast cancer and breast cancer prevention

A two-tailed matched pairs *t*-test was run to find if there was a difference in the mean presurvey and postsurvey knowledge scores for all eligible participants (postsurvey minus presurvey score). The *p*-value of <0.001, with *t*(199) = 3.996, indicated that there is a statistically significant difference between presurvey and postsurvey scores. The mean difference was 0.792 with a standard error of the mean of 0.198.

### Hypothesis 3: increase in knowledge of breast cancer and breast cancer prevention by educational intervention group

The ANOVA test examined if there was a statistically significant difference in survey scores (postsurvey minus presurvey) among intervention groups. Contrary to the third hypothesis, there is insufficient evidence to suggest that there is a difference in knowledge change based on the intervention strategy [*F*(16, 183) = 1.477, *p* = 0.112].

### Hypothesis 4: factors predictive of screening mammography outcome

Bivariate relationships were examined using Kendall's Tau correlation. Weak, positive relationships with screening mammography that are statistically significant are distance to screening mammography site (*r* = 0.115, *p* = 0.02) and health insurance status (*r* = 0.213, *p* = 0.001). Weak, negative relationships that are statistically significant with screening mammography are age (*r* = −0.117, *p* = 0.03) and the number of years lived in the United States (*r* = −0.118, *p* = 0.032). In addition, weak/moderately weak and positive relationships that are statistically significant with screening mammography include preawareness (*r* = 0.136, *p* = 0.001), English proficiency (*r* = 0.146, *p* = 0.002), birthplace (*r* = 0.130, *p* = 0.016), and education (*r* = 0.280, *p* = 0.001).

Table 4 summarizes the results of a binary logistic model with mammography adherence as the dependent variable. All variables from Tables 1 and 2 are included in the mode1, except for postsurvey score as the postsurvey was completed after the mammography appointment date had passed. In addition, presurvey and postsurvey scores were highly correlated (Pearson correlation *r* = 0.371, *p*-value = 0) with each other. Therefore, removing postsurvey improved the reliability of the model by reducing confounding effects. The model is statistically significant (*p* = 0.039) compared to the null model; ∼21.1% of the variability within mammography adherence can be explained by the predictive factors.

**Table 4.** Binary Logistic Regression of Predicting Mammography Adherence

	Overall		
	Odds ratio	SE	Significance
Constant	0.078	3.14	0.417
How far away is the mammography site from your home? (miles)	1.016	0.017	0.369
Employment status			
I am in school	0	40,192.97	1
Unemployed >12 months	0.948	0.483	0.912
Unemployed ≤12 months	0.495	0.926	0.447
Part-time employed	0.585	0.445	0.228
Full-time employed	^a^		0.69
Age	0.978	0.027	0.409
Highest education degree			
No education	18.226	0.89	0.001
Elementary	19.918	0.885	0.001
High School	11.332	0.817	0.003
College/Grad	^a^		0.008
English communication and proficiency	1.085	0.051	0.106
Is there a place where you often go when you feel sick or need help?			
No	0.429	1.398	0.544
Do you have access to transportation?			
Yes	1.404	0.416	0.414
How much do you think you know about “Affordable Care?”			
A lot	0.328	0.97	0.25
More or less	0.505	0.546	0.211
Little	1.389	0.429	0.445
Nothing	^a^		0.168
In general, how would you describe your health?			
Excellent	1.588	1.019	0.65
Good	1.09	0.625	0.89
Somewhat good	0.842	0.61	0.778
Poor	^a^		0.874
How many years have you lived in the United States?	0.971	0.028	0.295
Where did you receive information about breast cancer prevention?			
Doctor	1.247	0.497	0.657
Family/friends	1.174	0.583	0.784
Media	0.878	0.529	0.805
Church	10.544	0.842	0.005
Other	^a^		0.053
In general, how would you rate the medical or health quality you have received?			
Excellent	1.6	0.662	0.478
Good	1.423	0.613	0.566
Regular	1.178	0.661	0.805
Poor	4.424	1.162	0.201
No health care received	^a^		0.744
Do you have health insurance?			
Yes	0.207	0.553	0.004
Presurvey score	1.145	0.083	0.102
Where were you born?			
Central America	1.472	1.628	0.812
South America	1.769	1.625	0.725
Mexico	0.75	1.711	0.867
Dominican Republic	^a^		0.655
Educational intervention strategy			
Cell phone messages	0.57	0.434	0.196
Text messages	0.557	0.445	0.189
Mail messages	^a^		0.326

		Chi-square	Significance
	Omnibus test of model coefficients	49.89	0.039
	Cox and Snell *R* square	0.211	

^a^ Note that excluded variables are the reference variables.

SE, standard error.

The statistically significant variables are degree of education (“no education” OR = 18.226, *p* = 0.001; “elementary education” OR = 19.918, *p* = 0.001; and “high school education” OR = 11.332, *p* = 0.003), “church for the place where they received breast cancer information” (OR = 10.544, *p* = 0.005), and health insurance status (OR = 0.207, *p* = 0.004).

The results of a binary logistic model with mammography adherence as the dependent variable stratified by intervention strategy reveal that the full model within the phone group is statistically significant (*p* < 0.001) compared to its null model; ∼74.5% of the variability within mammography adherence can be explained by the predictive factors (data not shown in Table). None of the predisposing factors is statistically significant within the phone model. The full model within the mail category is not different from its null model (*p* = 0.139); ∼41.8% of the variability within mammography adherence can be explained by the predictive factors.

The statistically significant variables are employment status (“I am in school” OR = 0.026, *p* = 0.050 and “unemployed ≤12 months” OR = 0.027, *p* = 0.021), “Affordable Care Act knowledge,” that is, knowledge level of the provisions of the Affordable Care Act (“More or less” OR = 0.061, *p* = 0.092), where breast cancer information was received (“church” OR = 2,662.374, *p* = 0.064), and health insurance status (OR = 0.072, *p* = 0.091). In the text group, ∼22.8% of the variability within mammography adherence can be explained by the predictive factors, which is not different from the null model (*p* = 0.956). The English index—communication and proficiency—is also statistically significant (OR = 1.2, *p* = 0.093) (data not shown in Table).

Table 5 summarizes the results of a multiple regression model with presurvey knowledge as the response variable. All variables from Tables 1 and 2 are included in the model, except for postsurvey score and mammography adherence as both occurred after the presurvey. Approximately 9.9% of the variability in the model is explained by the factors included (adjusted *R*-square = 0.099). Having “a lot” of knowledge about the Affordable Care Act (standardized estimate = 0.128, *p* = 0.088) and not having any education (standardized estimate = −0.376, *p* = 0.017) are statistically significant variables in the model.

**Table 5.** Multiple Regression of Predicting Presurvey Score of Breast Cancer Knowledge

	Overall		
	B	SE	Significance
Constant		2.609	0.002
How far away is the mammography site from your home? (miles)	−0.029	0.016	0.703
Employment status			
I am in school	0.075	2.428	0.296
Unemployed >12 months	−0.047	0.447	0.596
Unemployed ≤12 months	0.01	0.818	0.893
Part-time employed	−0.059	0.429	0.509
Full-time employed	^a^		
Age	0.134	0.024	0.106
Highest education degree			
No education	−0.376	0.74	0.017
Elementary	−0.087	0.753	0.521
High school	0.034	0.702	0.806
College/Grad	^a^		
English communication and proficiency	0.035	0.046	0.702
Is there a place where you often go when you feel sick or need help?			
No	−0.02	1.205	0.78
Do you have access to transportation?			
Yes	0.013	0.394	0.86
How much do you think you know about “Affordable Care?”			
A lot	0.128	0.97	0.088
More or less	0.054	0.5	0.536
Little	−0.083	0.391	0.313
Nothing	^a^		
In general, how would you describe your health?			
Excellent	−0.056	0.886	0.506
Good	0.083	0.582	0.498
Somewhat good	0.039	0.568	0.749
Poor	^a^		
How many years have you lived in the United States?	−0.051	0.026	0.584
Where did you receive information about breast cancer prevention?			
Doctor	−0.003	0.473	0.977
Family/friends	0.101	0.551	0.246
Media	−0.005	0.502	0.956
Church	−0.128	0.685	0.163
Other	^a^		
In general, how would you rate the medical or health quality you have received?			
Excellent	0.151	0.619	0.166
Good	0.074	0.577	0.548
Regular	0.177	0.616	0.107
Poor	0.1	1.042	0.215
No health care received	^a^		
Do you have health insurance?			
Yes	−0.029	0.504	0.721
Where were you born?			
Central America	−0.39	1.263	0.125
South America	−0.366	1.267	0.102
Mexico	−0.204	1.373	0.228
Dominican Republic	^a^		
Educational intervention strategy			
Cell phone messages	0.075	0.403	0.353
Text messages	0.089	0.409	0.286
Mail messages	^a^		
	Correlation coefficient (*r*)	0.49	
	Adjusted *R*-square	0.099	

^a^ Note that excluded variables are the reference variables.

A multiple regression model for presurvey score stratified by educational intervention group demonstrates that for the phone group, unemployment for >12 months is the only statistically significant variable (standardized estimate = 0.35, *p* = 0.07) (data not shown in Table). For the mail group, unemployment for >12 months is statistically significant (standardized estimate = −0.306, *p* = 0.092). The model for the mail group explains the variance within presurvey knowledge better than the model for the phone and the text groups—∼3.6% of the variance is explained by the individual-level factors (adjusted *R*-square = 0.036) (data not shown in Table).

## Discussion

The results of this study revealed that the percentage of mammography adherence did not differ among the three educational intervention groups, and there was no statistically significant difference in knowledge of breast cancer and breast cancer prevention among the groups. Therefore, contrary to hypotheses, all three intervention strategies are equally effective in contributing to the increase in knowledge and mammography adherence. Although these results are not statistically significant, they are practically significant. The results provide insights on a “best practice patient-centered model” to enhance cancer prevention efforts among an underserved population.

One plausible explanation for the results is that the actual method of communication (whether phone messages or text messages) or the usual care (mail) is not what ultimately matters in increasing screening mammography rates and breast cancer knowledge among Latina immigrants. The key issue in promoting breast cancer knowledge and the uptake of screening mammography among Latinas is *how* the messages are constructed and *who* the messenger is, rather than which communication method is adopted to deliver the messages.

The automated educational messages sent to all the participants, regardless of method of communication, were personalized, linguistically and culturally tailored, and provided relevant and clear information to the audience, purposefully targeting information gaps related to breast cancer and prevention. In addition, the educational messages in the intervention incorporated a faith- or family-based component as these elements were identified as essential in the development of the message during the formative focus groups.

For the study participants, it is also important to note the role of religion in health as a potential explanation for the high overall rate of mammography adherence. Previous studies document the role of religiosity as an enabling factor for preventive health behaviors such as cancer screenings among ethnically diverse populations.^23–25^

These results are consistent with previous studies that demonstrate, for cancer prevention messages to be effective among Latinas, the messages must resonate among members of the group and must be culturally appropriate.^26^ The messages were also sent by a credible and trustworthy source of information—a patient navigator from *Nueva Vida*, which increases the likelihood of building the rapport and trust needed to draw women to the messages.^26^

Another potential explanation for the nonsignificant results in this study is that for all the intervention groups, the usual barriers to screening mammography encountered by the population of low-income, mostly uninsured Latina immigrants were removed. Previous studies have documented barriers such as lack of health insurance, cost of a mammogram, inflexible work schedules, lack of transportation, and lack of interpreters.^8–12^

In this study, free mammograms were offered to the participants during flexible hours (*i.e.*, after-work and weekend hours), and free transportation on Saturdays to the screening mammography sites was guaranteed for all participants who needed it. All these benefits were offered as part of the intervention study, which likely served as strong facilitators for the uptake of screening mammography and breast cancer knowledge, regardless of means of communication, among this low-income, mostly uninsured population.

Education mattered substantially in predicting mammography adherence. Preawareness also impacted mammography adherence, which was expected because we would assume that those who know more about breast cancer would take the opportunity to get a free mammography. Church as a source of breast cancer information was very important as well, which is consistent with the literature.^23–25^ On the other hand, health insurance status was not an important predictive factor. Free mammogram screenings may have impacted the statistical significance of health insurance status in the model.

This study has several limitations. First, the survey questions included a “Not Sure” option, which did not properly measure if the individual knew the answer. Second, some data were missing for various predictive factors. Third, small sample sizes for the study groups did not allow us to build plausible models. Also, we did not have enough participants born in Mexico or the Dominican Republic and thus could not conduct stratified regressions.

Future research that increases the sample sizes for individuals' birthplaces would allow for the data to be stratified by region. In addition, examining the dependence of one explanatory variable on another explanatory variable through interaction terms may provide further insight into mammography adherence. It would also be useful to conduct a cost-effectiveness study of the intervention strategies by assessing both indirect and direct costs for each strategy, the labor hours per screening, and the phone and text messaging service costs per screening for each intervention group.

There are substantial opportunities for community health centers and clinics to reduce disparities in breast cancer and breast cancer screening. Based on the results of this study, the key to increasing breast cancer knowledge and screening adherence among underserved, ethnically diverse populations lies in the provision of culturally and linguistically tailored breast cancer and screening education delivered by a patient navigator or other trusted source of information. A breast cancer preventive intervention program that uses the individuals' preferred method of communication and is combined with a free or low-cost mammogram holds promise to be an effective method in reaching underserved populations with high breast cancer burdens.

## Abbreviations Used

ACS: American Cancer Society
ANOVA: analysis of variance
mHealth: mobile health
N/A: no answer
OR: odds ratio
RCT: randomized control trial
SD: standard deviation
SE: standard error
USPSTF: US Preventive Services Task Force
